# Machine learning models in predicting graft survival in kidney transplantation: meta-analysis

**DOI:** 10.1093/bjsopen/zrad011

**Published:** 2023-03-29

**Authors:** Bharadhwaj Ravindhran, Pankaj Chandak, Nicole Schafer, Kaushal Kundalia, Woochan Hwang, Savvas Antoniadis, Usman Haroon, Rhana Hassan Zakri

**Affiliations:** Department of Renal Transplantation, Guy’s and St Thomas’ NHS Foundation Trust, London, UK; Department of Renal Transplantation, Guy’s and St Thomas’ NHS Foundation Trust, London, UK; Centre for Nephrology, Urology and Transplantation, King’s College London, London, UK; Department of Renal Transplantation, Guy’s and St Thomas’ NHS Foundation Trust, London, UK; Department of Renal Transplantation, Guy’s and St Thomas’ NHS Foundation Trust, London, UK; Department of Renal Transplantation, Guy’s and St Thomas’ NHS Foundation Trust, London, UK; Department of Renal Transplantation, Guy’s and St Thomas’ NHS Foundation Trust, London, UK; Department of Renal Transplantation, Guy’s and St Thomas’ NHS Foundation Trust, London, UK; Department of Renal Transplantation, Guy’s and St Thomas’ NHS Foundation Trust, London, UK; Centre for Nephrology, Urology and Transplantation, King’s College London, London, UK

## Abstract

**Background:**

The variations in outcome and frequent occurrence of kidney allograft failure continue to pose important clinical and research challenges despite recent advances in kidney transplantation. The aim of this systematic review was to examine the current application of machine learning models in kidney transplantation and perform a meta-analysis of these models in the prediction of graft survival.

**Methods:**

This review was registered with the PROSPERO database (CRD42021247469) and all peer-reviewed original articles that reported machine learning model-based prediction of graft survival were included. Quality assessment was performed by the criteria defined by Qiao and risk-of-bias assessment was performed using the PROBAST tool. The diagnostic performance of the meta-analysis was assessed by a meta-analysis of the area under the receiver operating characteristic curve and a hierarchical summary receiver operating characteristic plot.

**Results:**

A total of 31 studies met the inclusion criteria for the review and 27 studies were included in the meta-analysis. Twenty-nine different machine learning models were used to predict graft survival in the included studies. Nine studies compared the predictive performance of machine learning models with traditional regression methods. Five studies had a high risk of bias and three studies had an unclear risk of bias. The area under the hierarchical summary receiver operating characteristic curve was 0.82 and the summary sensitivity and specificity of machine learning-based models were 0.81 (95 per cent c.i. 0.76 to 0.86) and 0.81 (95 per cent c.i. 0.74 to 0.86) respectively for the overall model. The diagnostic odds ratio for the overall model was 18.24 (95 per cent c.i. 11.00 to 30.16) and 29.27 (95 per cent c.i. 13.22 to 44.46) based on the sensitivity analyses.

**Conclusion:**

Prediction models using machine learning methods may improve the prediction of outcomes after kidney transplantation by the integration of the vast amounts of non-linear data.

## Teaser

This systematic review explores the current application of machine learning models and performs a meta-analysis of these models in the prediction of graft survival after kidney transplantation. We aim to summarize the current available evidence, identify the best machine learning models suited for these outcomes, and the key challenges that need to be addressed to accurately guide future research in this topic. Prediction models using machine learning methods can better predict outcomes after kidney transplantation by the integration of the vast amounts of non-linear data.

## Introduction

Artificial intelligence (AI) consists of computerized algorithms designed to mimic and elaborate human thought patterns or actions. Machine learning (ML), one of the major branches of AI, is the study of algorithms that learn from sample data or past experience without being specifically programmed to perform a particular task^1^. ML techniques are progressively being applied in many disciplines to solve clinical and health-related problems^2^. The global market value of AI/ML has been predicted to grow from 4.3 billion Euros in 2020 to 42.4 billion Euros by 2026^3^. This is due to ML/AI’s ability to swiftly analyse large amounts of complex and non-linear data, act as a potential adjunct for clinical diagnosis, and accurately predict outcomes compared with traditional statistical methods^4,5^.

The four commonly used methods in ML are supervised, unsupervised, semi-supervised, and reinforcement learning^6,7^. The most common supervised learning methods such as neural networks and classification-based models recognize patterns in the training data set and help make predictions by identifying similar patterns in future data sets. In contrast, unsupervised models aim to identify hidden patterns in a data set and are not trained in a previous data set. Semi-supervised learning is a bridge between supervised and unsupervised learning that is trained using a smaller fraction of labelled data and a significantly larger set of unlabelled data. Reinforcement learning is a technique in which the algorithm automatically learns from feedback in a data set in a trial and error manner, thereby closely mimicking human learning.

Common ML models include neural-based models such as artificial neural networks (ANN), convolutional neural networks (CNN), decision trees (DT), random forest (RF) and support vector machines (SVM). Multiple hybrid models combining many aspects of these basic models have been developed and used in healthcare and are discussed in this review. Neural-based models such as ANN are inspired by neurons and contain ‘nodes’ that communicate with other nodes via connections based on their ability to perform a specific task. A CNN is a subtype of ANN that is predominantly used in image recognition algorithms as it preserves the spatial relationship between pixels in an image. A CNN relays parts of data to specific nodes with a view to preserve the spatial orientation of the feature extracted^8,9^.

A DT is a non-parametric supervised learning technique used for classification tasks. It is similar to a flow chart, starting from a root node and splitting into multiple branches and nodes. Each node represents a test on a particular attribute, each branch represents the outcome, and the terminal node holds the class label. An RF is an extension of this where an ensemble method produces multiple DT^10^.

Despite the advances in kidney transplantation (KT), the accurate prediction of graft survival (GS) after transplantation using standard statistical modelling continues to be a challenge^11–14^. Existing risk prediction models such as donor–recipient pairing or the kidney donor risk index (KDRI) have a limited ability to predict outcomes for kidney transplant recipients with receiver operating characteristic (ROC) scores of 0.6–0.7^14–16^. Although ML has been used to predict GS and various other outcomes after solid organ transplantation, there is significant inconsistency regarding the accuracy and effectiveness of these prediction models^17–20^.

The purpose of this paper is to systematically review and determine the current status of AI/ML models in the prediction of GS after KT.

## Methods

### Search strategy

The authors aimed to identify all published studies in which ML models were used to predict GS after KT. They searched the MEDLINE, Elton Bryson Stephens Company Information (EBSCO) and Embase databases from their earliest date until 14 November 2022 by using pre-specified key words (*Supplementary material*). Article screening and extraction was performed using the Covidence online screening and data tool^21^. The reference lists of the retrieved articles and similar review articles in the field were also searched to identify additional papers. All studies written in English and focusing on the clinical prediction of GS after renal transplantation by ML-based models were included. Case reports, non-English papers, editorials/commentaries, conference abstracts, pre-print articles, reviews, letters, and papers with limited data on methodology were excluded. The study was registered in the PROSPERO database (CRD42021247469) and was performed according to PRISMA guidelines^22^.

### Data extraction

The key details regarding the method and results were recorded on a data extraction sheet. Data extraction was conducted by two independent reviewers (B.R. and N.S.). Discrepancies were resolved by discussion amongst the authors and a tiebreaking vote from the authors not involved in the screening process (K.K., U.H., and P.C.).

Data elements extracted included study name and year of publication, country, method of feature selection, ML method used, validation methods, study population, the type of input variables (pre-transplant, intraoperative, and/or post-transplant), size of the training and validation data sets, results, and follow-up interval.

### Quality and risk-of-bias assessment

The methodological quality of the studies included in the review was assessed using the AI/ML-specific quality assessment tool introduced by Qiao^23^. This instrument proposes the following categories: unmet need or limits in current non-ML approach, robustness, reproducibility, generalizability, and clinical significance. The risk-of-bias assessment of the studies was carried out using the PROBAST tool^24^. The risk-of-bias assessment and quality assessment figures were produced with the help of the interactive online web application, ‘robvis’^25^.

### Meta-analysis

The suitability of pooled analyses was considered via interpretation of heterogeneity based on the *I*^2^ statistic and *P* value for the χ2 test. Given the significant heterogeneity in the included studies, ML models, methodology, and the index test used to evaluate ML model performance, the single point estimate for the overall model was calculated by meta-analysis of the area under the ROC (AUROC) curve, calculation of summary estimates of sensitivity and specificity, and subsequent construction of a hierarchical summary ROC (HSROC) curve. The performance of the ML-based models and regression-based models in the prediction of short-term (less than 1 year) GS and long-term (greater than 3 years) GS were analysed. The calculations were based on the random effects bivariate binomial model of Chu and Cole^26^ and the HSROC curve parameters were calculated based on the equations drawn from Harbord *et al.*^27^. The HSROC curve was constructed using the online MetaDTA tool^28,29^ and the meta-analysis of the AUROC curve was performed based on the method outlined by Zhou *et al.*^30^. A sensitivity analyses was performed that included studies with no significant methodological concerns and low risk of bias and studies that validated their data set in a separate or external data set.

## Results

**Fig. 1:**
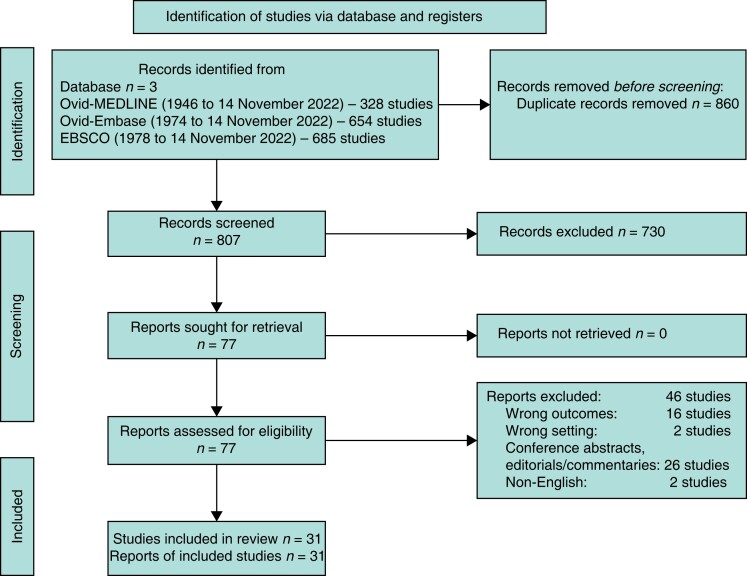
Flow chart of study inclusion as per PRISMA guidelines EBSCO, Elton Bryson Stephens Company Information Services.

**Fig. 2:**
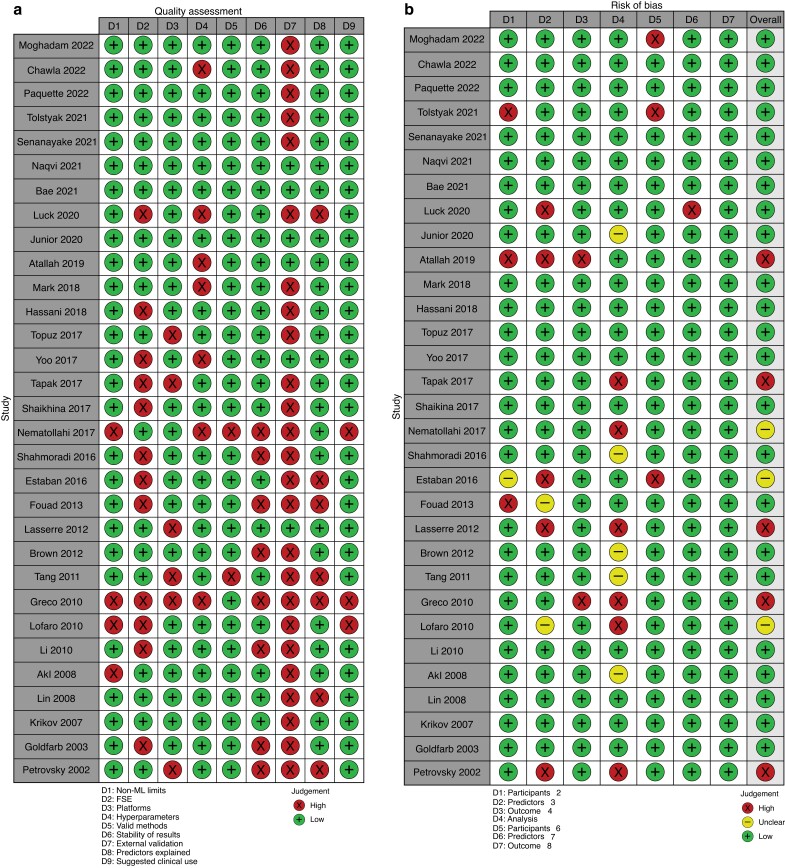
a Summary of the quality assessment by the criteria described by Qiao^23^. b Summary of the risk-of-bias assessment based on the PROBAST tool ML, machine learning; FSE, feature selection engineering.

Out of the 1667 studies identified, 31 studies met the inclusion criteria for the systematic review. The exclusion of all the other studies is outlined in *Fig. 1* in accordance with the PRISMA reporting guidelines. The quality assessment of these studies as assessed by the criteria set by Qiao^23^ revealed that 12 studies did not perform feature selection engineering (FSE), only five studies validated the model in an external data set, and eight studies reported an instability of result (*Fig. 2a*). Eighteen studies, however, used a separate subset of data to validate their models. The risk-of-bias assessment using the PROBAST tool indicated that five studies had a high risk of bias and three studies had an unclear risk of bias (*Fig. 2b*).

Most studies included a large number of preoperative donor and recipient input variables. Nineteen studies used FSE to identify the most relevant clinical variables prior to modelling. The studies in the review used 29 different ML methods including ANN, recurrent neural networks (RNN), DT, SVM, Bayesian belief networks (BBN), gradient boost (GB), adaptive boosting, and various hybrid models to develop their predictive models. Fourteen studies used more than one method to develop the models in their paper. Nine studies compared the performance of regression-based models versus ML-based models. A summary of all the included studies is included in *Table 1*.

**Table 1:** Characteristics of the included studies

Study	*n*	Study cohort	Feature selection method	Training and testing method and sets	ML algorithm	Outcome of interest	Index test	Model performance
Moghadam 2022^31^	378	NS	Red Deer clustering algorithm	10-Fold CV	Supervised: SVM, ANN, KNN, DT and Ensemble	Graft failure (NS)	Accuracy Sensitivity Specificity F1 score AUC	Accuracy: SVM: 0.94, ANN: 0.86, KNN: 0.92, DT: 0.96, Ens: 0.89 Sensitivity: SVM: 88%, ANN: 53%, KNN: 88%, DT: 94%, Ens: 65% Specificity: SVM: 96%, ANN: 98%, KNN: 94%, DT: 97%, Ens: 98% F-1 score: SVM: 0.87, ANN: 0.57, KNN: 0.85, DT: 0.92, Ens: 0.61 AUC: SVM: 0.95, ANN: 0.95, KNN: 0.97, DT: 0.95, Ens: 0.95
Chawla 2022^32^	5155	Single centre (NS)	Information gain African buffalo optimization	70/30	Supervised: AB-ANN	Graft failure	Precision Accuracy F measure Recall	97.6% 99.89% 98.2% 99.2%
Paquette 2022^33^	210 688	SRTR DD	Lasso CV Elastic net CV RFE	5-Fold CV	Supervised: Deepsurv Deephit RSF RNN	Death censored GS	C-index IBS ICI 1 y ICI 5 y ICI 15 y	C index: Deepsurv: 0.65, Deephit: 0.661, RSF: 0.64, RNN: 0.659 IBS: Deepsurv: 0.15361, Deephit: 0.1525, RSF: 0.15288, RNN: 0.15220 ICI 1yr: Deepsurv: 0.00957, Deephit: 0.01171 RSF: 0.01058, RNN: 0.00989 ICI 5y: Deepsurv: 0.0099, Deephit: 0.0285, RSF: 0.0174, RNN: 0.0107 ICI 15y: Deepsurv: 0.012, Deephit: 0.135, RSF: 0.0456, RNN: 0.0263
Tolstyak 2021^34^	164	NS	Information gain Correlation Boruta FS and RFE	70/30; 10-fold CV	Supervised: DF, LR, Adaboost, MLP, KNN, RF, SVM Polynomial, SVM Radial, SVM Linear, Ensembles: Boosted RF<LR, CARD, Stacking	Early graft failure	Accuracy Sensitivity Specificity	DF: Acc: 0.8, Sens: 0.78, Spec: 0.74 Adaboost: Acc: 0.84, Sens: 0.8, Spec: 0.8 MLP: Acc: 0.68, Sens: 0.62, Spec: 0.56 KNN: Acc: 0.81, Sens: 0.8, Spec: 0.74 RF: Acc: 0.84, Sens: 0.82, Spec: 0.77 SVM Polynomial: Acc: 0.82, Sens: 0.8, Spec: 0.79 SVM Radial: Acc: 0.78, Sens: 0.8, Spec: 0.8 SVM Linear: Acc: 0.78, Sens: 0.78, Spec: 0.78 Boosted RF: Acc: 0.82, Sens: 0.82, Spec: 0.76 GB: Acc: 0.85, Sens: 0.87, Spec: 0.79 CART: Acc: 0.83, Sens: 0.8, Spec: 0.8 Stacking: Acc: 0.9, Sens: 0.94, Spec: 0.86
Senanayake 2021^35^	7365	ANZDATA DD	Expert opinion Principal component analysis Elastic net	70/30	Supervised: Survival tree, Random survival forest, Survival SVM	DCGF	C-index	RSF: 0.63 SVM: 0.62 DT: 0.60
Naqvi 2021^36^	52 827	SRTR DD & LD	Paired features One-hot encoding Stacked auto-encoders	10-Fold CV	Supervised: SVM, AB, RF, ANN, LR Unsupervised Stack encoders used for FSE	GS 1y GS 5y GS 5–17 y	AUROC AUROC	SVM 0.82; AB 0.78; RF 0.70; ANN 0.61; LR 0.62 SVM 0.66; AB 0.69; RF 0.65; ANN 0.63; LR: 0.62 SVM 0.80; AB 0.81; RF 0.75; ANN 0.72; LR: 0.69
Bae 2020^37^	133 431	SRTR DD & LD	Literature review Exploratory data analysis Univariable models using different sets of knots	70/30	Supervised: Regression, GB, RF	DCGF 5 y ACGF 5 y	C-index C-index	Regression 0.637; GB 0.642; RF 0.638 Regression 0.634; GB 0.635; RF 0.633
Luck 2020^38^	131 709	SRTR DD	One-hot encoding unit scaling	80/20	Supervised: Traditional Cox MLP	GS	C-index	Cox: 0.650; MLP combined: 0.655
Junior 2020^39^	118	Single centre DD & LD	LR; Multivariable analysis	NS	Supervised: LR in an ML model	GS 1 y	AUROC	0.70
Atallah 2019^40^	2728	Single centre DD & LD	IGBFS; NBBFS	K-fold CV 70/30	Supervised: J48, NB, MLP, RF, SVM (IGBFS-NBBFS/KNN)	GS	Sensitivity F1 score	Sensitivity: J48 68.7, NB 60.6, MLP 70.4, RF 75.9, SVM 76.9, (IGBFS-NBBFS/KNN) 80.4 F1 score: J48 66.9, NB 63, MLP 69.1, RF 68.5, SVM 67.2, (IGBFS-NBBFS/KNN) 72.9
Mark 2019^41^	100 000	UNOS DD & LD	Cox Lasso	CV: Harrell’s C-index Integrated Brier score	Supervised: RF with CI trees	GS 5 y	C-index	0.724
Hassani 2018^42^	156	Single centre NS	Under and over sampling	75/25	Supervised: MLP RBF Binary particles swarm optimization & KNN	GS 2 y	Accuracy Precision Senstivity Specificity	MLP: Acc: 81.5, Precision: 83.5; Sens: 88; Spec: 62.5 RBF: Acc: 92, Precision: 92.85; Sens: 92; Spec: 71.4 BPSO & KNN: Acc: 96.87, Precision: 89.6; Sens: 100; Spec: 89.6
Yoo 2017^43^	3117	Multicentre DD & LD	NS	80/20	Supervised: DT	GS 10 y	C-index	0.71
Nematollahi 2017^17^	717	Single centre NS	Current evidence	NS	Supervised: SVM, ANN	GS 5 y	Accuracy Sensitivity Specificity AUC	SVM: 85.9%; ANN: 90.4% 97.3%; 98.2% 26.1%; 49.6% 0.769; 0.865
Topuz 2018^44^	31 207	UNOS LD, DD	Literature review Elastic net SVM, ANN Bootstrap forest combined with sensitivity analysis and info fusion	CV	Supervised: BBN	GS <3 y GS 3–7 y GS >7 y Incl.GS	Accuracy Sensitivity Specificity	Accuracy GS <3 years: 71% Accuracy GS 3-7 years: 74% Accuracy GS>7 years: 59% Accuracy Overall GS: 68% Overall Sensitivity: 41% Overall specificity: 84%
Tapak 2017^20^	378	Single centre LD, DD	NS	70/30	Supervised: ANN	GS	AUROC Sensitivity Specificity	0.88 91% 74%
Shaikina 2017^19^	80	HLA incompatible DD, LD	NS	75/25	Supervised: DT, RF	ABMR at 30 days	Accuracy Sensitivity Specificity AUROC	DT 85%, RF 85% DT 81.8%; RF 92.3% DT 88.9%; RF 71.4% DT 0.854; RF 0.819
Shahmoradi 2016^18^	513	Single centre LD, DD	NS	70/30	Supervised: ANN, C5, C&RTree	GS Overall	Sensitivity Specificity Accuracy	ANN: 87.1%; C5 90.8%; C&RTree 86.8% ANN: 65%; C5 52%; C&RTree 57.3% ANN: 83.7%; C5 87.2%; C&RTree 83.3%
Esteban 2016^45^	2061	NS	NS	NS	Supervised: GRU + static LSTM + static RNN + static TLE, LR	GS <1 y	AUROC	GRU: 0.833; LSTM: 0.826; RNN: 0.822; TLE: 0.821; LR: 0.808
Fouad 2015^46^	1900	Single centre LD	NS	10-Fold CV	Supervised: M5Rules REPTree LR	GS 5 y	Correlation coefficient	M5Rules: 0.87; REPTree: 0.73; LR: 0.733
Lasserre 2012^47^	707	Euro-transplant data DD	RFE	CV	Supervised: G-SVM	GS 1 y	AUROC Sensitivity Specificity	0.72 51.6% 86%
Brown 2012^48^	7348	USRDS DD	Current evidence	70/30	Supervised: NBB	GS 1 y GS 3 y	AUROC Sensitivity Specificity	AUROC (GS 1 year): 0.63 Sensitivity (GS 1 year): 39.9% Specificity (GS 1 year): 79.9% AUROC (GS 3 year): 0.59 Sensitivity (GS 3 year): 39.8% Specificity (GS 3 year): 80.2%
Tang 2011^49^	4754	USRDS SLE	Current evidence	10-Fold CV	Supervised: LR TBM ANN	GS 3 y	AUROC	Long list of predictors: LR: 0.74; TBM: 0.7; ANN: 0.71 Short list of predictors: LR: 0.73; TBM: 0.7; ANN: 0.70
Greco 2010^50^	194	Single centre LD, DD	NS	CV	Supervised: DT	GS 5 y	Sensitivity Specificity	Sensitivity: 88.2% Specificity: 73.8%
Lofaro 2010^44^	80	Single centre LD, DD	NS	CV	Supervised: DT	GS 5 y	Sensitivity Specificity AUC	62.5% 92.8% 0.847
Li 2010^51^	1228	UNOS	NS	5-Fold CV	Supervised: BBN	GS <1 y GS 1–5 y GS 5–10 y GS >10 y	Sensitivity Specificity AUROC	GS <1 y: Sn 85.8%, Sp 95.7%, AUC 0.967 GS 1–5 y: Sn 63.8%, Sp 88%, AUC 0.866 GS 5–10 y: Sn 54.2%; Sp 88%; AUC 0.866 GS >10 y: Sn 64.6%, Sp 89%, AUC 0.856
Akl 2008^52^	1900	Single centre LD	Univariable analysis	83/17	Supervised: ANN	GS 5 y	Sensitivity Specificity AUROC	88.4% 73.2% 0.88
Lin 2008^53^	57 383	UNOS LD, DD	Current evidence	CV	Supervised: ANN	GS 1 y GS 3 y GS 5 y GS 7 y	AUROC	0.73 0.75 0.77 0.82
Krikov 2007^54^	92 844	USRDS LD, DD	Multiple LR Survival analysis	66/34	Supervised: Tree based	GS 1 y GS 3 y GS 5 y GS 7 y GS 10 y	AUROC	0.626 0.640 0.717 0.830 0.901
Goldfarb Rumyantzev 2003^55^	37 407	UNOS DD	NS	70/30	Supervised: ANN	GS 3 y	(r) PPV NPV	0.984 76% 53.8%
Petrovsky 2002^56^	1542	UNOS DD	NS	NS	Supervised: ANN	GS 1 y	Accuracy	59%

SRTR, scientific registry of transplant recipients; DD, deceased donor; LD, living donor; GB, gradient boosting; RF, random forest; C-index, concordance index; CV, cross-validation; SVM, support vector machines; ANN, artificial neural networks; FS, feature selection; FSE, feature selection engineering; GS, graft survival; AUROC, area under the receiver operating characteristic; AB, adaptive boost; LR, logistic regression; MLP, multilayer perceptron; IGBFS, information gain based feature selector; NBBFS, naïve Bayes-based feature selector algorithm; CART, classification and regression tree algorithm; NB, naïve Bayes; KNN, K-nearest neighbour; DT, decision trees; NS, not specified; HLA, human leucocyte antigens; ABMR, antibody-mediated rejection; GRU, gated recurrent unit; LSTM, long-short term memory; RNN, recurrent neural networks; RSF, random survival forest; TLE, two-line element; G-SVM, gamma support vector machines; RFE, recursive factor elimination; USRDS, United States Renal Data System; TBM, tree-based model; UNOS, United Network for Organ Sharing; BBN, Bayesian-belief networks; PPV, positive predictive value; NPV, negative predictive value; ANZDATA, Australia and New Zealand Dialysis and Transplant Registry; DCGF, death censored graft failure; ACGF, all-cause graft failure; AUC, area under the curve.

The data from primarily deceased donor transplantation were used in seven studies and the data from exclusively living donor transplantation were used in two studies. All the other papers included data from both living and deceased donor transplantation. Fifteen studies used data from national or international registries such as the United States Renal Data System (USRDS), the United Network for Organ Sharing (UNOS), the Australia and New Zealand Dialysis and Transplant Registry (ANZDATA), or Euro-transplant data.

There was a substantial heterogeneity with respect to the study methodology, sample size, input variables, ML model performance, and the index test used to assess model performance. The main outcome measures used to assess the performance of ML models were AUROC curve, sensitivity, specificity, accuracy, and concordance index (C-index). Twelve studies evaluated their model performance mainly using sensitivity, specificity, and accuracy. The data from these studies were analysed to perform an HSROC curve analysis and the calculation of the summary estimates of sensitivity and specificity. Eighteen studies evaluated their model performance using ROC curve analysis. Seventeen studies used ML-based models to predict GS beyond 3 years, 11 studies used ML models to predict GS at 1 year or less and three studies did not mention the time interval.

**Fig. 3:**
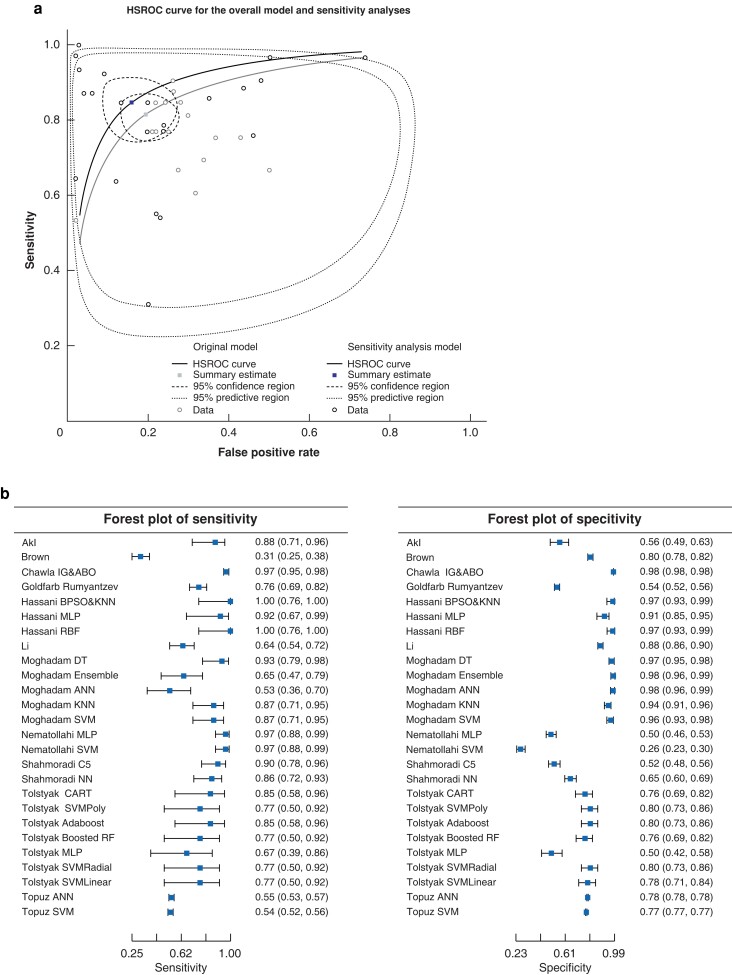
a Hierarchical summary receiver operating characteristic curve of the studies reporting sensitivity and specificity of machine learning model performance (overall model and sensitivity analyses). b Forest plots of sensitivity and specificity based on the sensitivity analyses including studies with no methodological concerns and with a low risk of bias. Values are left side: sensitivity (95% c.i.); right side: specificity (95% c.i.) HSROC, hierarchical summary receiver operating characteristic; IG&ABO, information gain African buffalo optimization; BPSO, binary particles swarm optimization; KNN, K-nearest neighbour; MLP, multilayer perceptron; RBF, random Bayesian forest; DT, decision trees; ANN, artificial neural networks; SVM, support vector machines; NN, neural networks; CART, classification and regression tree; SVMPoly, support vector machines with polynomial; Adaboost, adaptive boost; RF, random forest.

**Fig. 4:**
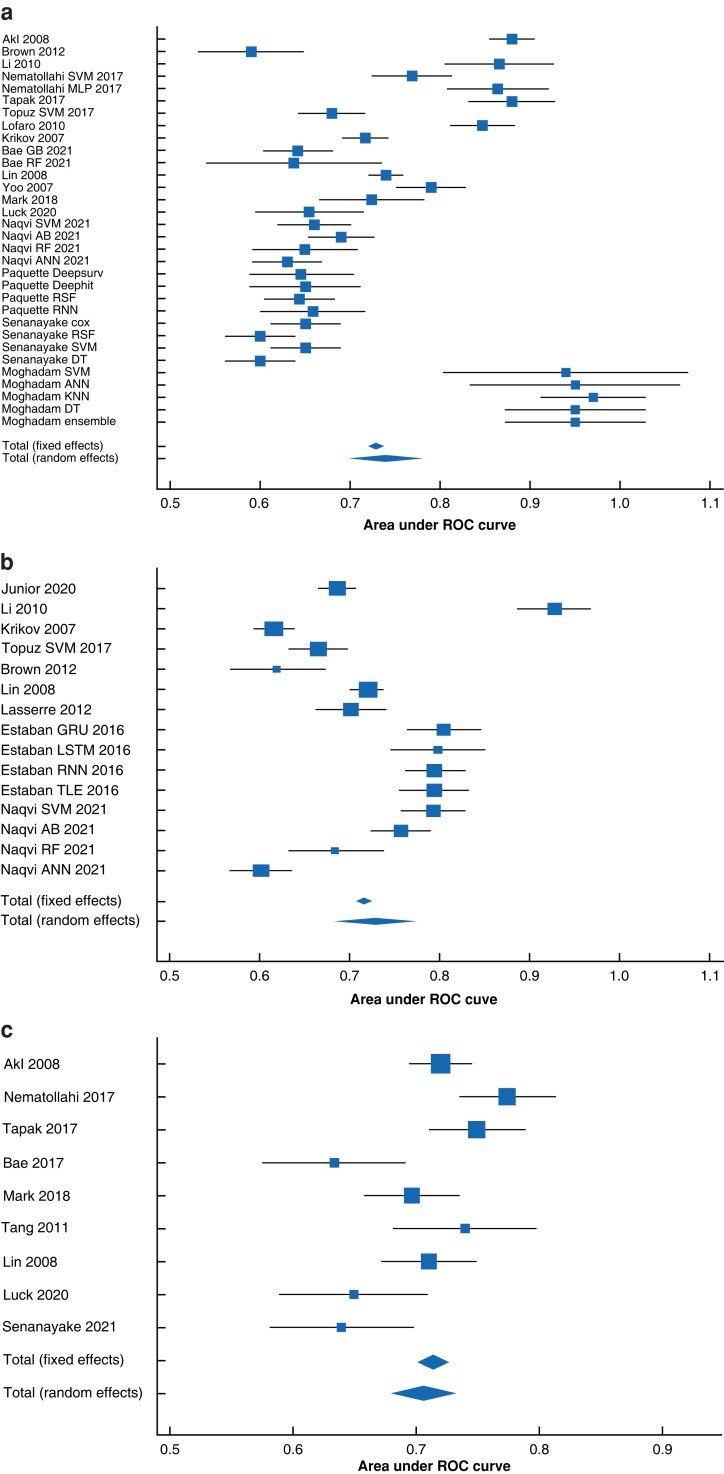
a Meta-analysis of the area under the receiver operating characteristic curve of machine learning model performance in the prediction of long-term graft survival. b Meta-analysis of the area under the receiver operating characteristic curve of machine learning model performance in the prediction of short-term graft survival. c Meta-analysis of the area under the receiver operating characteristic curve of regression model performance in the prediction of long-term graft survival SVM, support vector machines; MLP, multilayer perceptron; GB, gradient boost; RF, random forest; AB, adaptive boost; ANN, Artificial neural networks; Deepsurv, deep feed-forward neural network survival algorithm; Deephit, a deep learning framework based on neural network approach to survival analyses; RSF, random survival forest; RNN, recurrent neural networks; DT, decision trees; KNN, K-nearest neighbour; GRU, gated recurrent units; LSTM, long-short term memory; TLE, two-line element; ROC, receiver operating characteristic.

The area under the HSROC curve was 0.82 for all the studies and 0.85 based on the sensitivity analyses solely including the studies of good methodological quality, low risk of bias, and validation of data in a separate data set (*Fig. 3*). The summary sensitivity and specificity of the overall model were 0.81 (95 per cent c.i. 0.76 to 0.86) and 0.81 (95 per cent c.i. 0.74 to 0.86) (*Fig. 3b*) respectively. The diagnostic odds ratio for the overall model was 18.24 (95 per cent c.i. 11.00 to 30.16) and 29.27 (95 per cent c.i. 13.22 to 44.46) based on the sensitivity analyses. A meta-analysis of the AUROC curve based on the calculation of weighted summary AUROC curve curve under the random effects model of Zhou *et al.*^30^ revealed an overall AUROC curve of 0.74 (95 per cent c.i. 0.69 to 0.78) for ML-based GS prediction at 3 years or more and 0.75 (95 per cent c.i. 0.7 to 0.8) for ML-based GS prediction at 1 year and 0.71 (95 per cent c.i. 0.68 to 0.74) for regression-based models (*Fig. 4*). The meta-analysis of the AUROC curve revealed that ML-based models performed marginally better than regression-based models.

## Discussion

This systematic review aims to summarize the current evidence surrounding the predictive ability of ML models in graft outcomes after KT. A total of 31 studies were included in the review and meta-analysis out of which approximately one-sixth of the included studies had a high risk of bias and 14 studies had some methodological concerns. The predictive ability of 29 different supervised ML models were evaluated in this review and significant heterogeneity was noted in the included studies with respect to the methodology and models used. Despite these limitations, ML-based models had a significantly higher HSROC curve, a higher diagnostic odds ratio, and AUROC curve of 0.82, which has thus far not been achieved by many traditional statistical models^37^.

Several attempts have been made to use ML-based algorithms to predict long-term GS. These attempts have included the use of DT, ANN, SVM, and BBN^56–59^. However, the best ML method used to develop a suitable model to predict outcomes after KT continues to be controversial and a widely discussed topic^17–19,43,60,61^. Innovative approaches to data mining could potentially improve the accuracy of these outcomes of organ transplantation by considering the non-linear association between the various factors^54,55,62^. These predictive models are limited by various factors including the reliance on pre-transplant clinical parameters, such as age, BMI, cold or warm ischaemia time, and type of dialysis; variable assessment of immunological factors; smaller sample sizes used to build the models; and the complex and non-linear interrelationship and failure to accurately use censored patient data.

In this review the authors noted significant heterogeneity in many domains of the ML models used, including the method of FSE, the non-ML limits, the platforms used to generate these models, and the index test used to predict model performance. There was a significant divergence with respect to the clinical data used including the number of patients in each study and the type of variables used to construct the ML models. The majority of studies used preoperative donor and recipient variables and very few studies incorporated intraoperative and postoperative predictors to construct the ML model. Many studies aimed to predict GS at 1, 3, or 5 years after transplantation and also reported a significant difference in the ability of these models to predict GS at various time points as indicated by the prediction intervals^63^. Only one study explored the use of ML-based models in prediction of the ‘time to event’^43^.

Although the best ML model to predict graft outcomes continues to be controversial, the results of this review suggest that hybrid ML models, variations of SVM, and RF-based models performed the best in the prediction of GS. There was a significant difference in the performance of ANN and tree-based models in the prediction of GS. Despite the reported heterogeneity in performance among the ML models, their overall and individual model performance is reportedly better than the prediction ability of the currently available gold-standard prediction tools such as the KDRI. The reported C-index of KDRI is 0.63^14,64^ and most ML models in this review report a better performance in the prediction of GS.

There is limited evidence available regarding the minimum sample size required to develop a sound ML-based predictive model. Although it has been noted in this review that ML models developed using both single-centre small-scale data and large-volume database data have reported similar predictive abilities, larger sample sizes resulted in better model performance. It is also noteworthy that model performance is highly dependent on the volume of clinical data and their linear or non-linear relationship, the complexity of the model, and the ML method used. It has also been noted that a higher number of events per variable was associated with better model stability and higher predictive accuracy^65^.

The predictive accuracy of the ML model used also depends on the integration of clinically significant variables in the model^66^. Hence, identification of the relevant clinical variables that need to be incorporated into the model is a key step in model development. FSE is a common and well recognized method to identify these variables and is critical to avoid overfitting. Overfitting occurs when too many clinical variables are incorporated into the model, which triggers the model to adapt to irrelevant details, eventually leading to poor predictive performance^67^. Twenty-six studies have used cross-validation or its variations to circumvent this problem. Cross-validation is the best tool in assessing the effectiveness of a model, particularly in cases that need to mitigate overfitting. It also helps in determining the hyper parameters of the ML model, to achieve the lowest test error. To generalize that AI/ML has applicability in the field of KT and is reliable at predicting GS, external validation of these ML models is crucial because ML models perform very well in cohorts in which they have been trained^68^. However, many published prediction models are either not externally validated at all or poorly externally validated^69^.

Cox models and logistic regression are less suited to handling complex or non-linear relationships between predictors/covariates and outcomes as they assume that variables are independent of each other^53,70,71^. Thus, the accurate prediction of complex outcomes such as allograft survival using these statistical techniques continues to be a challenge^72^. The currently available NHS Blood and Transplant (NHSBT) risk prediction tool is modelled based on traditional statistical models with limited preoperative donor and recipient variables. The ideal prediction model should ideally include preoperative donor and recipient variables, intraoperative variables, and postoperative variables to accurately predict GS. ML models trained on data-sets with a large amount of clinically irrelevant data can result in unintended biases^73^. Representative biases can occur in clinical databases or genetic databases and can be potential pitfalls irrespective of the type of ML method used. ML models trained on retrospective/historical data sets may not necessarily reflect current practice and therefore model testing on large data sets with clinically relevant variables is vital for good predictive performance^74,75–79^.

Limitations of this review include the methodological shortcomings arising out of the substantial heterogeneity within the included studies and the difficulty in arriving at a single summary estimate of overall model performance. The evidence presented in this review has also suggested that most model predictions are based on a very large amount of retrospective data with limited external validation. In an ideal ML setting, a specific prospective data entry based on clinical experience should be combined with graft outcome data and then analysed over an interval of time in conjunction with actual outcomes. The most recent study comparing the predictive abilities of ML models *versus* conventional statistical models performed in a large database has suggested that AI/ML models are not significantly superior to conventional regression-based models^37^. Although the authors agree that no substitute can replace human intelligence or clinical experience, the results of this review have demonstrated that the best prediction of difficult outcomes such as GS, which takes into account numerous preoperative, operative, and postoperative outcomes, is difficult with human intelligence alone. An informed and well guided decision is best taken by combining clinical experience and a well designed prediction model. These well designed prediction models can only be developed by ML tools given the vast amounts of data required to build a reliable model. This review summarizes the current available evidence, identifies the best ML models suited for these outcomes, and the key challenges that need to be addressed to accurately guide future research. Whilst the use of AI/ML in KT is still in its infancy, such models have a significant future role, not only in the prediction of GS, but, the authors believe, also in organ matching, diagnostics, and management pathways.

## Supplementary Material

zrad011_Supplementary_Data

## Data Availability

The template data collection forms, data extracted from included studies and data used for all analyses are available from the corresponding author (B.R.) upon reasonable request.
